# Cellulite and the Aesthetic Management of the Buttocks and Thighs: 6 Cases Illustrating Targeted Verifiable Subcision as Part of a Multimodal Approach to Lower Body Rejuvenation

**DOI:** 10.1093/asjof/ojae031

**Published:** 2024-04-30

**Authors:** Laurie A Casas, M Bradley Calobrace, Johnny Franco, Jennifer Harrington, Kristi Hustak, Sachin M Shridharani

## Abstract

**Background:**

In the buttocks and thighs, skin quality, focal adiposity, volume deficiency, skin laxity, and/or textural issues each contribute to overall appearance. For patients undergoing rejuvenation/beautification procedures, global improvement is desired, making multimodal treatment the standard of care to address these mechanistically distinct concerns. Resolution of cellulite depressions is central to patient satisfaction and aesthetic outcomes: without management, the overall aesthetic suffers, and patients are left partially unsatisfied with treatment results. With minimally invasive Targeted Verifiable Subcision (TVS; Avéli [Revelle Aesthetics, Inc., Mountain View, CA]), septa with a confirmed role in dimple formation can be released through mechanically verified subcision, permitting consistent outcomes.

**Objectives:**

Discuss the application of TVS as part of a multimodal approach to buttock and thigh rejuvenation and share best practices for obtaining optimal improvement.

**Methods:**

A group of 6 experts in aesthetic plastic surgery and dermatology convened for a 2 h roundtable discussion of select case studies, best practices, and their approaches for obtaining optimal outcomes in clinical practice.

**Results:**

Clinical cases from 6 patients who presented for buttock and/or thigh rejuvenation/beautification are presented where TVS was applied as part of a multimodal approach. Before and after images, details of patient cases, and a discussion of best practices for patient education and evaluation, treatment planning, technique, safety, postprocedure care, and open research questions are included.

**Conclusions:**

TVS is emerging as a valuable tool for the treatment of cellulite in the buttocks and thighs that may potentially be used alongside surgical and nonsurgical approaches, often on the same day.

**Level of Evidence: 4:**

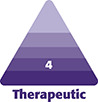

Historically, lower body rejuvenation and beautification have been challenging areas of aesthetic medicine and cosmetic surgery due to the difficulty inherent in achieving a balanced, global improvement in the buttocks and thighs, as well as the limitations of any single device or intervention for achieving global improvement. In the buttocks and thighs, issues such as adiposity, volume deficiency, skin laxity, and textural/topographical issues such as cellulite each contribute to overall appearance. More often than not, these issues occur together; however, due to different underlying etiologies, multiple mechanistically distinct interventions are required to achieve global improvement that will satisfy the patient.

For the buttocks and thighs, whether being managed in a surgical or nonsurgical setting, cellulite is central to overall aesthetic: if left unmanaged, overall aesthetic suffers, and patients are left partially unsatisfied with the results of their treatment. Importantly, it is not just the aesthetic result that is compromised—there are everyday, quality-of-life consequences. Cellulite can affect multiple aspects of everyday life, from the patient's choice of clothing to self-perception and confidence,^1,2^ and often, these feelings are the very reasons patients are seeking care.^3^ Similarly, cellulite managed in isolation is generally insufficient, as patients are often left with skin laxity or focal volume deficiencies or excesses that detract from the overall aesthetic. Thus, the resolution of cellulite dimples should always be considered as a part of lower body rejuvenation/beautification.

In the cases presented here, the authors have incorporated Targeted Verifiable Subcision (TVS; Avéli [Revelle Aesthetics, Inc., Mountain View, CA])^4^ for the treatment of cellulite dimples into their own unique approaches to lower body beautification/rejuvenation. The cases each incorporate multiple modalities, including liposuction with and without fat transfer and skin tightening with hyperdilute biostimulatory fillers and/or various energy-based devices, as well as topical skin care.^5,6^

## METHODS

On October 18, 2023, a group of 6 experts in aesthetic medicine and lower body rejuvenation convened for a 2 h roundtable, hosted by Premier Aesthetic Solutions, LLC, to discuss TVS as part of a multimodal approach to lower body rejuvenation. Prior to the meeting, each author submitted case studies for discussion, and the meeting itself was focused on discussion of select cases, best practices, approaches for obtaining optimal outcomes in clinical practice, and open questions/areas where evidence is needed to improve clinical care. Case studies and clinical pearls from accompanying discussions are presented here. This collection of case studies does not represent a formal study, and thus was not approved by an institutional review board (IRB). The patients shown were treated as part of standard clinical care and were not study participants. Each patient provided consent for treatment and written consent to publish their photographs, and all patients were treated according to the principles outlined in the Declaration of Helsinki.

TVS is currently cleared by the United States Food and Drug Administration for the long-term reduction of cellulite in the buttocks and thighs.^4^ Unlike technologies that require trauma to the skin at each individual dimple, TVS permits the minimally invasive release of multiple cellulite fibrous septal bands from a single or small number of transcutaneous entry points. Prior to the procedure, individual cellulite dimples are marked with the patient in the standing position (shown as circles in treatment marking images). At this stage, entry points and tracts for the device probe are also planned (shown as solid lines in several marking photographs). The device probe is 15 cm in length, so dimples to be treated from a single entry point must be within 15 cm. Anesthesia is administered along planned tracts as well as at the device entry point. At each entry point, the TVS probe is advanced subdermally in the subcutaneous layer to each depression, using a light near the end of the probe to confirm depth. Once the depression is reached, a small hook is deployed and used to pull each septal band, thereby confirming its contribution to individual dimple formation. Next, each band is cleaved, and the hook may be used to confirm that all contributing bands have been cut (Video). Efficacy and safety for TVS have been demonstrated for up to 12 months; however, mechanical subcision is presumed to be permanent.^7-10^ As in the cases discussed here, TVS can be used as part of nonsurgical or surgical treatment. The procedure for buttocks and posterior thigh typically takes the skilled clinician 20 to 60 min, and the authors have found that it is relatively easily integrated into workflows.

## RESULTS

**Figure 1.**
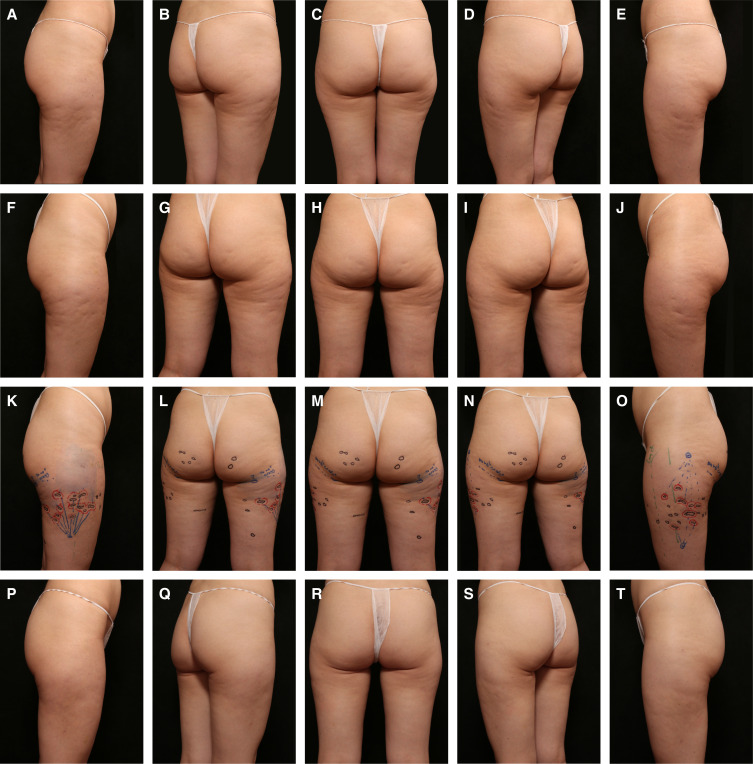
Case study 1. A 32-year-old female patient is shown at baseline (A-E), 6 weeks following the second of 2 rounds of CaHA/MFU-V treatment (F-J) at which time Targeted Verifiable Subcision (TVS) was carried out. Treatment markings for TVS are shown in K-O. The patient is shown 6 months after treatment with TVS (P-T).

**Figure 2.**
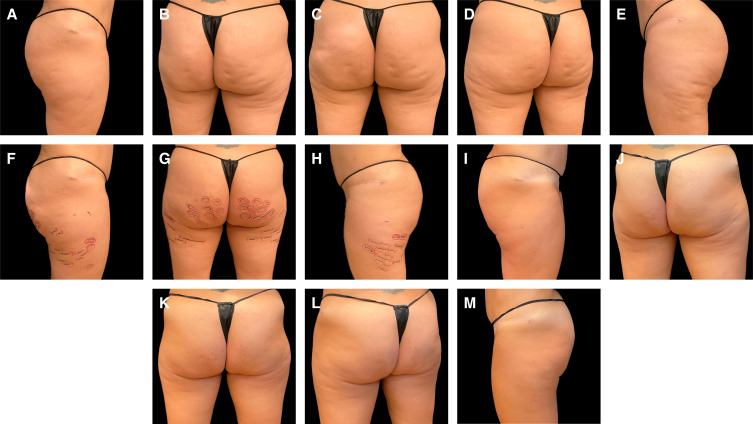
Case study 2. A 40-year-old female patient at baseline (A-E) with treatment plan markings for Targeted Verifiable Subcision (TVS) (F-H). The patient was treated with TVS, and beginning at 7 weeks following TVS was treated with radiofrequency microneedling, administered as 3 separate sessions, 4 and 8 weeks apart. The patient is shown 5 months following treatment with TVS (I-M).

**Figure 3.**
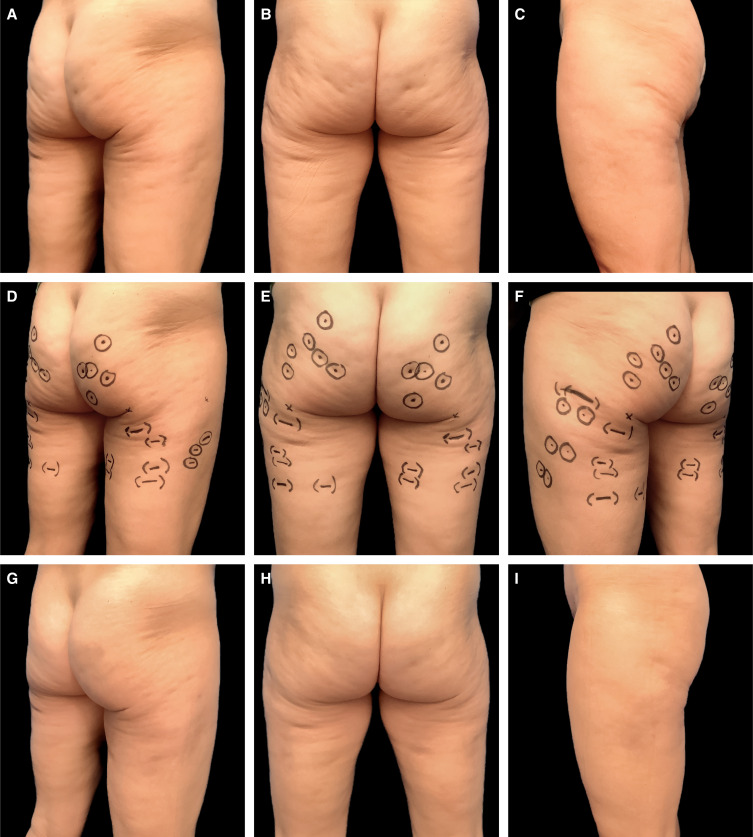
Case study 3. A 36-year-old female patient at baseline (A-C), with treatment markings for Targeted Verifiable Subcision (TVS) (D-F). The patient was treated with radiofrequency microneedling and TVS on the same day and is shown 6 months following treatment (G-I).

**Figure 4.**
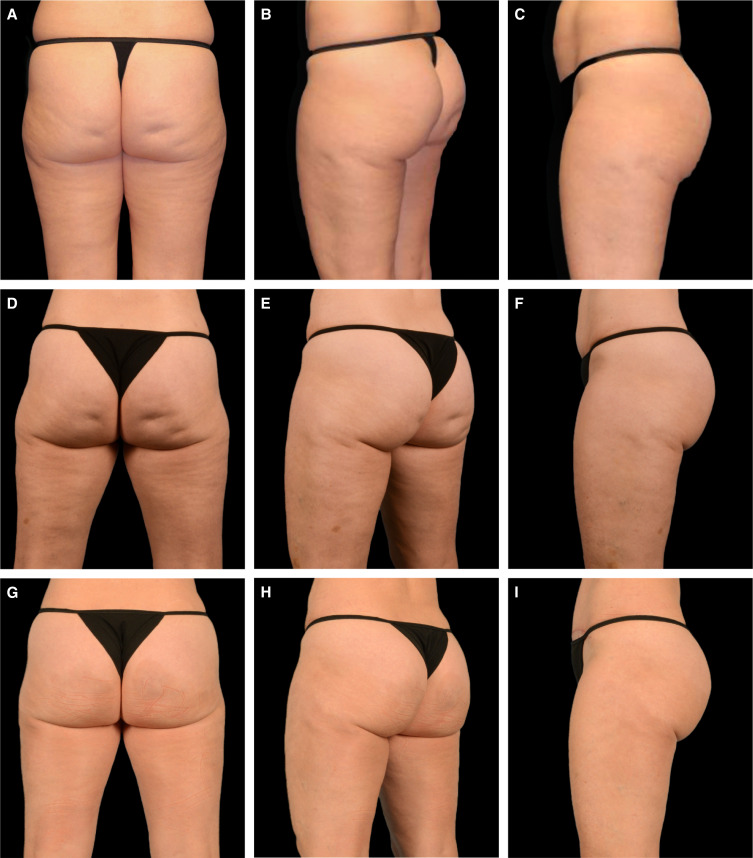

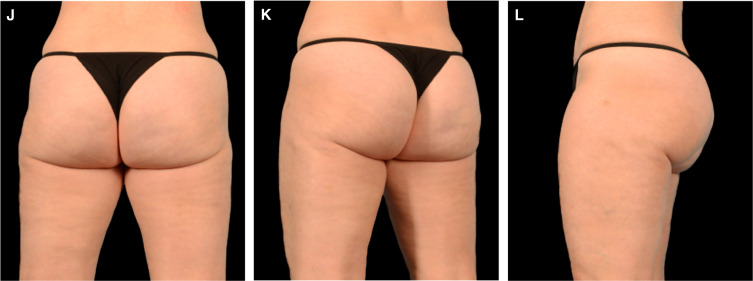
Case study 4. A 43-year-old female patient at baseline (A-C) and 6 weeks after liposuction and fat grafting (D-F). Ten months later, the patient received additional lipousuction, radiofrequency microneedling and Targeted Verifiable Subcision, performed on the same day, in that order. The patient is shown 7 weeks (G-I) and 1 year and 11 months postprocedure (J-L).

**Figure 5.**
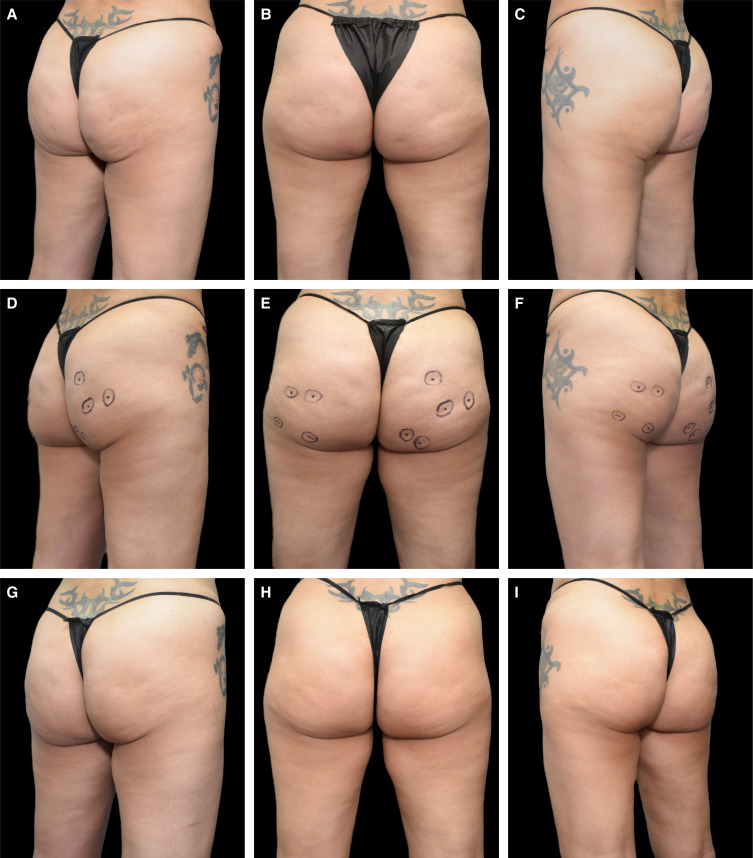
Case study 5. A 48-year-old female is shown after liposuction, fat grafting, and abdominoplasty (A-C). One year and 8 months following the initial procedure, the patient was treated with Targeted Verifiable Subcision (TVS) in the buttocks and posterior thighs (markings are shown in, D-F). The patient is shown 4 months after TVS (G-I).

Clinical cases from 6 patients who presented for buttock and/or thigh rejuvenation are presented in Figures 1 to 6. Patient information, treatment approaches, and additional case details are provided. Although in some of the presented cases, patients received lower body treatments in sequence over the course of months or years, authors are more likely to use TVS on the same day or within 6 to 8 weeks of initial treatment, as part of a lower body rejuvenation/beautification package or customized treatment plan. All contributing authors agreed that patients should be educated on the importance of improving and maintaining skin quality to best ensure long-term success in their lower body beautification restoration and rejuvenation.

### Case 1

A 32-year-old female presenting with posterior and outer thigh skin laxity and cellulite depressions (Figure 1A-E) underwent 2 rounds of skin tightening, 5.5 months apart, to the outer and posterior thighs with microfocused ultrasound with visualization (MFU-V; Ultherapy; Merz North America, Inc., Raleigh, NC; 150 lines, 3 and 4.5 mm). At these same treatment sessions, the patient also received 3 cc of calcium hydroxylapatite (CaHA; Radiesse; Merz North America, Inc.): 1.5 cc CaHA, diluted 1:2 with normal saline, to each posterior and lateral thigh, consistent with published guidelienes.^11^ The combination of MFU-V and hyperdilute CaHA^12^ was selected based on the complementary skin-tightening mechanisms and the positive results observed for this combination in clinical studies.^3^ Six weeks following the last CaHA/MFU-V treatment (Figure 1F-J), the patient underwent TVS to release cellulite depressions on her outer and posterior thighs (treatment markings are shown in Figure 1K-O). The patient opted to delay the release of her buttocks and some of her posterior thigh cellulite depressions until a later date.

At the time of her first MFU-V and CaHA treatment, the patient began twice-daily application of 2 products, a topical body treatment with tripeptide and hexapeptide (TransFORM Body Treatment with TriHex Technology; Alastin Skincare, Inc., Carlsbad, CA) and Regenerating Skin Nectar with TriHex Technology (Alastin Skincare, Inc.).^12^ Prior to treatment with TVS, the patient began using a topical treatment that combines the active agents in TransFORM Body Treatment and Regenerating Skin Nectar: ReFORM & RePAIR with TriHex Technology (Alastin Skincare, Inc.), used twice daily for the 3 weeks prior to and 12 weeks following the procedure to improve postprocedure healing.^5^

The patient is shown 6 months after TVS in Figure 1P-T. For TVS, there were 2 entry points per side, one on the gluteal fold and the other on the outer thigh. By placing the TVS probe entry point on the outer thigh, all dimples on the thigh could be reached from a single incision. Placement of this incision below the dimples allowed for drainage of the injected local anesthetic following the treatment and in the authors’ experience can reduce the risk of seroma. One element of this case to note is the deliberate inclusion of topical treatment on the skin of the treated areas. Although the necessity of proper skin care for the face is never questioned, skin care for the lower body is often neglected. In this case, and many others in this author's clinic, skin care of the body is an important part of optimizing the aesthetic both before and after treatment.

### Case 2

A 40-year-old female patient was treated for skin laxity and cellulite as part of a larger weight loss and rejuvenation plan. When the patient had lost 20 pounds and was halfway to her goal weight, her cellulite was treated. The patient is shown at baseline, just prior to TVS (Figure 2A-E) and with treatment plan markings for TVS (Figure 2F-H). The patient was treated with TVS, and after 7 weeks, the patient was treated with radiofrequency (RF) microneedling (Morpheus8; InMode, Inc., Irvine, CA), administered as 3 separate sessions, 4 and 8 weeks apart (Figure 2I-M). Settings were 2 and 3 mm in the buttocks and 4 and 5 mm in the thighs, based on skin thickness in the area, and energy was delivered at 25 to 30 mJ. Many patients within this author's practice receive rejuvenation/beautification treatments within the context of significant weight loss and therefore have substantial laxity issues. Although the patient presented here received treatment as a sequence, this author commonly administers TVS and RF microneedling on the same day, employing TVS first. TVS has also been safely used following fat transfer in this author's practice, using ultrasound guidance to ensure the probe remains in the correct plane. When laxity and cellulite are managed at the outset with combination treatments that address the multiple factors leading to patient aesthetic concerns, the patient is able to experience a more balanced and significant improvement in the treated areas. Furthermore, managing laxity and cellulite at the outset of the treatment course allows any risk of ptosis to be mitigated, and the patient is able to experience results sooner.

### Case 3

A 36-year-old female patient presented with significant laxity and cellulite (Figure 3A-C). The patient was treated with TVS (markings shown in Figure 3D-F) and RF microneedling (Genius; Lutronic Co., Korea) using the M49 dermal applicator on the same day, employing TVS first to release dimples. The goal for RF microneedling was to reach 40 to 60 mJ per pin, with the first pass at a depth of 2.5 mm and the second pass at a depth of 2 mm. The patient is shown 6 months following treatment (Figure 3G-I). This case is an important reminder that even patients with substantial laxity can be treated successfully. For this patient, improvement in both laxity and dimples was needed to improve surface irregularities and shadowing. This level of improvement in a nonsurgical setting was satisfying for the patient and illustrates the degree of improvement possible when laxity and cellulite are addressed together.

### Case 4

A 43-year-old female patient is pictured at baseline (Figure 4A-C) and after liposuction of calves, inner thighs, knees, abdomen, and outer thighs (750 cc/side) and fat grafting to the central buttocks (500 cc per side; Figure 4D-F). The patient underwent additional liposuction 10 months later to the outer thighs and knees (250 cc/side), treatment with RF microneedling (Morpheus8, 40-pin burst, 2-4 mm depth, 25-35 mJ) to the outer thighs, buttocks, and knees, followed by TVS to the buttocks and outer thighs that day, in that order (Figure 4G-I). Some slight bruising was apparent at 7 weeks; however, all discoloration dissipated without intervention by Week 12. Images of the patient 1 year following TVS are shown in Figure 4J-L.

### Case 5

A 48-year-old female presented for lower body rejuvenation. The patient is shown after liposuction to the upper abdomen, upper back, and flanks; fat grafting to the buttocks (400 cc transferred to the right buttock and 400 cc transferred to the left buttock); and abdominoplasty (Figure 5A-C). One year and 8 months following the initial procedure, the patient was treated with TVS in the buttocks and posterior thighs (markings are shown in Figure 4D-F). The patient is shown 4 months after TVS in Figure 4G-I. This case is an example of a common phenomenon: patients are often bothered more by cellulite following augmentation. In many of the authors’ practices, TVS is offered as part of gluteal augmentation or liposuction procedures to improve overall satisfaction and eliminate the need for a subsequent procedure to manage cellulite. In the authors’ experience, once patients are educated on the difference between laxity and cellulite, they often choose to add TVS to their treatment package.

### Summary of Results

Together, these case studies illustrate the versatility of TVS as well as the importance of cellulite resolution to the overall patient aesthetic. In the authors’ experience, cellulite must be managed in order for rejuvenation in the buttocks and thighs to reach its full potential. When reviewing the presented cases, one may note several remaining volume and contour issues; however, these cases represent highly satisfied patients. Conversely, patients for whom skin laxity, focal adipose volume excess or deficiencies, and buttock projection have been resolved are generally not fully satisfied when cellulite dimples remain, irrespective of whether or not cellulite was initially pinpointed as a key concern or aesthetic priority.

## DISCUSSION

Lower body rejuvenation and beautification require a multimodal, nonsurgical, and often surgical treatment plan. Every patient has a unique set of concerns, typically involving both the skin and subcutaneous fat layers. Cellulite on the buttocks and thighs is nearly universal in postpubescent biological females (present in 80%-90%^13^ of females^13-18^), and treating these dimples and depressions as well as managing skin changes and fat distribution are generally focal points of patients’ short- and long-term aesthetic plans.^19^ TVS provides an option for managing a condition that is both significant to patients and important for achieving optimal aesthetic outcomes in the buttocks and thighs. A summary of important considerations for patient evaluation, multimodal treatment planning and staging (ordering), TVS incision placement, and safety optimization are detailed in the sections below.

### Patient Evaluation

Patient education at the time of presentation is critical. It permits an opportunity not only to shape patient expectations but also to propose a short- and long-term treatment program designed to address the patient's leading concerns. For example, the patient in Case 1 was asked to pull her skin taut along her upper outer thigh area, and the treating clinician asked her if she would be satisfied with the type of skin laxity improvement observed, noting that improvement would be limited to unevenness in the skin and that cellulite dimples would not be resolved. In this case, the patient selected a treatment plan that would address both issues.

The visualization of cellulite during this process is key. It is important to ensure that patients are able to see their buttocks and thighs well during the evaluation process (eg, by standing in front of a full-length mirror) so that they can actively participate in treatment decisions. Standardized clinical photography is also critical, as it allows patients to be able to appreciate the different components of their concerns. This way, when a multimodal approach is planned, the patient better understands why each concern may need to be treated differently. With sequential photography, the patient can also see and appreciate gradual changes and the degree of improvement obtained along their lower extremity beautification journey.

### Patient Prioritization of Cellulite

Because cellulite is almost ubiquitous in biological females (present in over 80%-90% of patients^14^), it is a key factor shaping most patients’ perceptions of their buttocks and thighs. Importantly, for many patients, the experience of cellulite is at least partially social, and patients often focus on deficits that prevent them from looking their best in form-fitting or revealing clothing and may vocalize a desire to feel differently in social situations. Often, patients are less concerned about prominent hip contour changes like hip dips, flattening of the lower buttocks, or outer thigh contour irregularities from lipodystrophy than they are with the unevenness caused by skin laxity and cellulite depressions.

For example, the patient in Case 2 is a candidate for additional volume to further improve hip contour and medial projection, and the patient in Case 6 is a candidate for treatment that would address hip contour; however, these patients were satisfied with their results and did not feel that they needed additional interventions at that time (note that it is important to educate patients regarding the continuum of care that is needed to treat areas of concern when they appear or as they age). This experience is not uncommon, and the advisors agreed that patients are most often focused on laxity, cellulite, and/or focal adipose volume deficiencies or excess. For example, the patients in Cases 2 and 3 were most concerned about the cellulite changes that they could see through their clothes (patients will often refer to the “white pants test”).

### Management of Laxity

To date, there are no data to guide whether laxity should be managed before cellulite. Because skin laxity occurs in every person with age, albeit in some more than others, skin laxity and age-related changes in the skin's texture are ever-present issues that need to be addressed in both the short and long terms.^19,20^

In Case 1, laxity was managed prior to cellulite, and in general, the authors agreed that dimples release more easily when laxity is managed before cellulite. In Cases 3 and 4, cellulite was addressed at the same time as laxity, resulting in excellent global improvement as well as an efficient treatment course for the patient. In Cases 2 and 6, laxity was managed after cellulite dimple release.

Based on these cases and the authors’ clinical experience, there is no clear indicator that order has a significant effect on outcome; however, clinical studies are needed to determine whether there is an optimal treatment sequence and whether all of the applied treatments shown in the cases are required to achieve optimal outcomes.

Importantly, in patients with severe laxity, there is some concern that treatment with TVS first may increase the risk for ptosis of localized adiposity around the lower buttock and high outer thigh; however, this concern is largely restricted to patients with severe laxity and adjacent adiposity. Comanagement of these conditions in patients with substantial laxity (eg, Case 2) may mitigate this risk to some degree. Of note, some of the cases presented here overlapped with TVS regulatory approval, and the authors agreed that as they have gained more experience with the device, they are more likely to incorporate it into an initial cotreatment for laxity, volume excess, or deficiencies.

Addressing skin quality is not just relevant for laxity but also for managing the dermal thinning that can accompany cellulite dimples.^21^ Skin thinning can create the appearance of shadowing/unevenness and make deeper dimples appear as incompletely resolved or adjacent areas of thinner skin appear as new dimples. All authors agreed that managing skin quality has an important effect on overall outcomes and that there is a need to develop effective skin care regimens for body skin, as has been done for the face, neck, and chest.

TVS provides long-term improvement of cellulite dimples^9^; however, skin laxity from aging and gravity is ongoing and progressive, necessitating ongoing management.

### Targeted Verifiable Subcision (TVS) Incision Placement

Within TVS clinical trials, almost all incisions were placed in the gluteal crease. When dimples on the outer and lateral thighs are treated, this entry point has limitations. The TVS probe is 15 cm long and is generally unable to reach all outer and lateral thigh dimples from the gluteal crease. Incisions may also be placed on the thighs, as shown in Cases 1 to 3. Although it is possible to place incisions in the thighs above or below the dimples to be treated, placing the incisions inferior to the dimples allows fluid to drain following the procedure and, anecdotally, reduces the risk of seroma in cases in which clusters of dimples are being treated, as in Case 1. Incisions inferior to the dimples can be manually drained the day following the procedure. Should they occur, seromas must be drained and managed quickly with aspiration when needed. For the buttocks, all authors agreed that incisions should be placed within the gluteal fold.

### TVS in the Posterior Thigh

The structures within and 1 to 2 cm (immediately) below the gluteal fold are critical for supporting the buttocks. Therefore, the authors agreed that dimples in this zone should not be treated. There are no data or guidelines exploring the minimum distance from the gluteal fold at which release of fibrous bands in the posterior thigh is safe. The authors typically restrict TVS treatment to dimples greater than 2 to 3 cm from the gluteal fold. Some authors treat dimples in the posterior thigh closer or even within this zone, as long as they are lateral to the natural gluteal fold and the patient has minimal laxity; however, not all authors treat cellulite dimples in this area, due to the concern for resultant fat and skin ptosis and past experience with outpouching following treatment in this area.

In the posterior thighs, it is not uncommon for dimples to be oblong and somewhat longer (Figure 2F-H). The authors agreed that when treating these dimples, especially if they are near the gluteal fold, the entire linear depression is not released, typically alternating between treating 1 cm segments and leaving intervening 0.5 to 1 cm of skin untreated. When treating linear depressions, it is also important to leave some adhesions so that the supportive septal network is not undermined, which could compromise support and allow fluid to collect. There is a need for additional research in this area and evidence-based guidance on this topic.

### Fibrous Septa and Buttock Shape

While the septal network within the buttocks and thighs can give rise to cellulite, these same septa also maintain buttock shape and play an important role in balancing the containment and extrusion forces present at the subdermal junction.^22^ An awareness of this function is critical, and cellulite depressions should be treated in a way that does not undermine the structural stability of the skin or the capacity of the septal network to support fat containment by the skin and the superficial and deep fascia. For example, in some patients, there is some lower pole expansion in the buttocks following the release of septa with TVS in the buttocks. This can cause the lower buttock to appear rounder or cause a slight lengthening of the gluteal fold, which can be appreciated in Figure 2. This effect can be desirable but should be kept in mind as a potential consequence of treatment. The density of septal release within a given area must be carefully considered. In instances where dimples are large and close together, conservative treatment that leaves some attachments in place is prudent—if too many bands are cut, then there can be a flattening or “lake effect” in that area due to loss of structural support. To date, the authors have not had many seromas; however, it stands to reason that the release of larger contiguous areas would result in undermining the tissue and could create a seroma. This is another area where data are needed to inform best practices.

### Safety and Side-Effect Mitigation

Given the association of the fibrous bands that cause cellulite with blood vessels,^22-24^ bruising is an expected side effect. Risk with TVS is inherently reduced because the skin is not broken at each individual dimple, but bruising risk can be further reduced by using a careful local anesthetic infiltration technique and incorporating tranexamic acid (1000 mg/10 cc/L of the local anesthetic mixture) and epinephrine into the local anesthetic mixture.

All authors advise patients to stop taking any medications, including supplements, that reduce platelet function and inherent clotting ability (eg, blood thinners, omega-3 fatty acids, and non-steroidal anti inflammatory drugs). In the experience of the authors, bruising with TVS is far less severe than that reported for enzymatic treatments or other mechanical subcision methods, an experience consistent with the safety observed in TVS clinical studies.^9,10^ In nearly all cases, any residual bruising or hemosiderin staining is resolved by 9 months. In Case 4, the level of bruising apparent at 7 weeks is typical. Lastly, it is important to allow time for the local anesthetic mixture agents to take effect before initiating treatment.

### Recommendations for Aftercare

Patients are generally instructed to wear compression garments for weeks following treatment, with no vigorous lower extremity exercise for 2 weeks. Compression with padding is recommended for the first 1 to 5 days, after which tight leggings are sufficient. To reduce bruising, inflammation, posttraumatic swelling, any induration from the release of larger cellulite dimples, and the appearance of the 3.5 mm TVS entry-site incision, patients are instructed to use a topical tripeptide and hexapeptide formulation, Alastin ReFORM & RePAIR.^5^

To improve skin quality over the long term, patients may also use topical Alastin TransFORM Body Treatment. As with all aesthetic procedures, home care is an important aspect of maintaining results long term and should include basic best practices such as eating a high-quality diet and getting appropriate exercise, using topical skin care products, wearing light-compression clothing when standing for long periods, and sitting properly to avoid multilayer tissue trauma from firm chairs.

Importantly, the information presented here is based on anecdotal experience: there remain open questions that must be more systematically addressed with clinical research, including optimal treatment ordering for skin laxity, local adiposities, and volume deficiencies; the distance from the gluteal fold beyond which it is safe to release septal bands; and the number of confluent dimples/depressions that can be safely treated. As TVS use increases, it will be important to develop evidence-based best practices on these topics. One additional limitation of any case series is that it may not illustrate the full range of effects that occur with treatment, as the number of patients presented is small. Furthermore, we are not able to determine from these cases the impact of each individual treatment, especially when they are carried out within a single session, thus, these cases do not represent sufficient evidence to say that each of the applied treatments is required to achieve optimal results. Finally, each of these cases could benefit from longer follow-up, as shorter term results have inherent limitations.

## CONCLUSIONS

All authors agreed that TVS has emerged as a reliable treatment for cellulite dimples and depressions. Although data on combination treatments are needed, clinically, TVS has been incorporated into nonsurgical and surgical approaches to buttock and thigh rejuvenation, often administered on the same day as laxity treatments, fat transfer, and volume repletion with fillers. The side-effect profile is manageable, and the results thus far are consistent, features lacking in other cellulite devices. For patients seeking lower extremity beautification, cellulite management is central to achieving an optimal outcome, and patients are rarely satisfied with other procedures if dimples remain. Skin laxity should be addressed and treated along with cellulite dimples to ensure a satisfactory outcome.
